# Anthropometric Status and Nutritional Intake in Children (6–9 Years) in Valencia (Spain): The ANIVA Study

**DOI:** 10.3390/ijerph121215045

**Published:** 2015-12-18

**Authors:** María Morales-Suárez-Varela, Nuria Rubio-López, Candelaria Ruso, Agustín Llopis-Gonzalez, Elías Ruiz-Rojo, Maximino Redondo, Yolanda Pico

**Affiliations:** 1 Unit of Public Health, Hygiene and Environmental Health, Department of Preventive Medicine and Public Health, Food Science, Toxicology and Legal Medicine, University of Valencia, Valencia 46100, Spain; nrubiolopez@hotmail.com (N.R.-L.); candela.ruso@gmail.com (C.R.); agustin.llopis@uv.es (A.L.-G.); 2 CIBER Epidemiología y Salud Pública (CIBERESP), Madrid 28029, Spain; ruiz_eli@gva.es (E.R.-R.); yolanda.pico@uv.es (Y.P.); 3 Center for Advanced Research in Public Health (CSISP-FISABIO), Valencia 46010, Spain; 4 Dirección General de Salud Pública, Conselleria de Sanidad, Valencia 46010, Spain; 5 Biochemistry Departament, Agencia Sanitaria Costa del Sol, University of Malaga, Red de Investigación en Servicios de Salud en Enfermedades Crónicas (REDISSEC), Marbella 29603, Spain; mredondo@hcs.es; 6 Food and Environmental Safety Research Group, Faculty of Pharmacy, University of Valencia, Valencia 46100, Spain; 7 Research Center on Desertification (CIDE, UV-CSIC-GV), Carretera Moncada-Náquera, Moncada 46113, Spain

**Keywords:** child, dietary intake, anthropometric status, dietary recommendations

## Abstract

The aim of our study was to assess nutritional intake and anthropometric statuses in schoolchildren to subsequently determine nutritional adequacy with Spanish Dietary Reference Intake (DRIs). The ANIVA study, a descriptive cross-sectional study, was conducted in 710 schoolchildren (6–9 years) in 2013–2014 in Valencia (Spain). Children’s dietary intake was measured using 3-day food records, completed by parents. Anthropometric measures (weight and height) were measured according to international standards, and BMI-for-age was calculated and converted into z-scores by WHO-Anthro for age and sex. Nutrient adequacy was assessed using DRI based on estimated average requirement (EAR) or adequate intake (AI). Pearson’s chi-square and Student’s *t*-test were employed. Of our study group (47.61% boys, 52.39% girls), 53.1% were normoweight and the weight of 46.9% was inadequate; of these, 38.6% had excess body weight (19.6% overweight and 19.0% obesity). We found intakes were lower for biotin, fiber, fluoride, vitamin D (*p* < 0.016), zinc, iodine, vitamin E, folic acid, calcium and iron (*p* < 0.017), and higher for lipids, proteins and cholesterol. Our results identify better nutritional adequacy to Spanish recommendations in overweight children. Our findings suggest that nutritional intervention and educational strategies are needed to promote healthy eating in these children and nutritional adequacies.

## 1. Introduction

Adequate dietary intake is of vital importance for children’s growth and development, not only in physiological terms, but also in mental and behavioral ones [1,2,3]. Hence the importance of early intervention based on acquiring healthy eating habits because they persist in later life [4]. Both excessive and inadequate intake of energy or nutrients may have a detrimental influence on children’s health, and predispose to childhood obesity, dental caries, underachievement at school and lower self-esteem [5,6,7,8], and also to diseases like hypertension, atherosclerosis, obesity, osteoporosis and type 2 diabetes later in life. This means that the prevention of these diseases should start as early on as childhood [2].

Many developed countries are undergoing epidemiological and health transitions with rapid increases in the incidence of overweight, obesity and chronic diseases [9,10,11,12,13,14] across all age groups as a result of changes in dietary and physical activity patterns [13,15,16]. When nutritional intakes are not adjusted to Dietary Reference Intake (DRI), some malnutrition type may exist, as in energy-rich diets and with low intakes of vitamins and minerals, essential nutrients for the organism to adequate function, which affect children’s growth and development [17,18,19].

In the USA, the NHANES study [20] showed that ~30% of American children aged 6–19 years were overweight (≥95th percentile for age) or at risk of being overweight (≥85th percentile, but <95th percentile for age). Overweight rates have almost tripled since the first NHANES study (1971–1974) [20]. According to the 2012 Mexican National Survey of Health and Nutrition [21] 34.4% of Mexican children aged 5–11 years are obese [18]. In Spain, the enKid study [22] found that children aged 6–9 years presented an overweight prevalence of 30.5% (14.5% overweight and 15.9% obesity). The ALADDIN study [23] established that the overweight overall prevalence in children aged 6–9 years was 44.5% (26.2% overweight and 18.3% obesity). This means that one child in every two was overweight.

Usually, childhood is the key step for adopting and consolidating eating habits. This group has been one of the groups most widely influenced by food globalization [18] given the transformation of the current food model with a wider range of industrial food, salty snacks, more soft drinks, skipping breakfast, not eating plenty of fruit, vegetables, grains and drinking milk, and abandoning traditional cuisine [14,18,24]. Thus their nutritionally inadequate diet makes them more vulnerable [25]. In the year 2000, the Krece Plus test assessed the quality of the diet of Spanish children aged 4–14. The results showed that 20% needed to make major adjustments in their diet, and the diet of 51% needed to be sporadically modified. The authors also observed that vitamins A, D, E and B_6_, and of Ca and Mg were deficient in both genders, while only 25% were considered to benefit from a quality diet [26].

In Spain, children’s growth acts as a sensitive indicator of health status, and its monitoring and evaluation are routine tasks of primary care pediatricians. Thus children who grow well will likely have no relevant associated diseases [27]. At times apparently healthy children with normal height for their age, and even overweight ones, suffer masked malnutrition, which can affect the maximum bone and intellectual development potential [17].

The present study aimed to assess nutritional intake and anthropometric statuses in schoolchildren (6–9 years old) and to subsequently determine nutritional adequacy with Spanish Dietary Reference Intake (DRIs).

## 2. Samples and Methods

### 2.1. Participants

The Antropometria y Nutrición Infantil de Valencia (Valencian Anthropometry and Child Nutrition, ANIVA) study is a descriptive cross-sectional study that was conducted in schoolchildren aged 6–9 years who went to one of the eleven participating primary schools. The estimated number of subjects was over 700 according to a simple size calculation based on our preliminary data (Type I error: 0.05, power: 0.8). Children were selected by means of random cluster sampling in schools, and stratified by sex and type of school (*i.e.*, public *vs.* private). The latter factor was used as an approximate indicator of socio-economic status. Sampling was carried out in two stages: first, schools were selected from lists made available by the Regional Educational Authorities; second, classrooms and pupils were selected.

Data collection took place during academic year 2013–2014. The study was orally presented to the Consejo Escolar (Board of Governors) of each participating school. Following this, a letter was sent to the parents of all the children invited to participate in the study, which outlined the study goals and procedures, and secured their written authorization. The inclusion criteria were: (a) children aged 6–9 years; (b) children who studied primary education at one of the eleven selected schools; (c) parents or legal guardians had to agree about the child participating and give written informed consent. The exclusion criteria were: (a) clinical diagnosis of chronic disease with dietary prescription; (b) absence from school on the days arranged to take body weight and height measures; (c) not properly completing the nutritional record.

The initial sample included 873 children of both genders, of whom 12.8% did not want to participate (*N* = 112). The subjects who provided incomplete information, improperly completed registration (*N* = 37) or did not present anthropometric measurements (*N* = 14) were removed. The participation rate was 81.3% and the resulting final sample comprised 710 children. The study protocol complied with Helsinki Declaration Guidelines and was approved by the Secretaría Autonómica de Educación, Conserjería de Educación, Cultura y Deporte of the Generalitat Valenciana, Valencia, Spain (2014/29630).

### 2.2. Anthropometric Measurements

During school hours, children’s height and weight were recorded by the same person following standard procedures described by the World Health Organization (WHO) [28], with children standing barefoot in light clothing. All the anthropometric measurements were obtained in duplicate and averaged.

Weight was measured to the nearest 0.05 kg using a calibrated electronic load cell digital scale (OMRON BF511^®^, Tokyo, Japan) and height was measured with a stadiometer (Seca 213^®^, Hamburg, Germany). Following the GPC Recommendations of the Spanish Ministry of Health and Social Policy, we took BMI as an index to calibrate nutritional status as it is an easy measure to obtain, is efficient and has been adopted internationally as a reasonable indicator of subcutaneous fat accumulation [29]. With these data, we calculated BMI-for-aged (z-score) with the Anthro software, v.3.2 (WHO, Geneva, Switzerland) [30,31]. Based on the obtained percentile ranking, BMI was used to classify children into one of the following four categories [31]: underweight (≤5th percentile), normoweight (>5th to <85th percentiles), overweight (≥85th to <95th percentiles) or obese (≥95th percentile).

### 2.3. Examination Protocol and Measurements

Parents were interviewed using a questionnaire to elicit information on their child’s age, sex, medical history, medication, and use of vitamin and mineral supplements. At the same time, we provided parents/guardians details of how to assess the food and drinks consumed by their child. They were asked to record estimated portion sizes for each ingested item. The same training was given to the caregivers responsible for the children while in school dining halls. A visual guide was provided to improve the accuracy of portion size estimates. This was essential to obtain reliable data. Parents were asked to submit food labels with ingredients, brands, added ingredients and the recipes for homemade dishes whenever possible. They were given a telephone number for information and support, which they could call to help resolve any issues that arose while completing the diary.

### 2.4. Dietary Assessment

To carry out the dietary survey, parents were asked to record all the foods and drinks consumed by their child over a 3-day period, including one non-working day (e.g., Sunday or Saturday) [32,33]. To calculate intakes of calories and macro- and micronutrients of known public health relevance, the researchers inputted data from the food records into an open-source computer software. This program (DIAL^®^, v2.16) [34], developed by the Department of Nutrition and Dietetics at the Madrid Complutense University, has been previously validated in Spain to assess diets and to manage nutritional data.

This open software includes a list of some of the enriched/fortified foods commonly available in Spain, to which other items can be added. It is possible to add foods to the database and, in this way, we were able to include the nutritional composition of packaged foods taken from food labels.

### 2.5. Estimate of Nutrient Adequacy/Deficiency

Dietary Reference Intakes (DRIs) [35,36,37] include values for Recommended Dietary Allowances (RDAs), Estimated Average Requirements (EARs), Adequate Intakes (AIs), and Tolerable Upper Intake Levels (ULs), as well as Estimated Energy Requirements (EERs) for energy, and Acceptable Macronutrient Distribution Ranges (AMDRs) for macronutrients. For each nutrient, children were categorized as being at risk of inadequate intake based on whether, or not, they met the corresponding nutritional targets [38] and DRIs [39] proposed for the Spanish population. Comparisons were made with the DRISs used in the USA to explore possible differences. The probability of adequate and usual intake of a given nutrient was calculated as follows: z-score = (estimated nutrient intake—EAR)/SD of EAR [40]. We used EARs for micronutrients, whenever available, and we took the AI values for the nutrients for which EARs were not determined (fiber, fluoride, manganese, potassium and pantothenic acid). The percentage of energy provided by proteins, lipids and carbohydrate was also calculated and compared with AMDRs. Using the data collected on consumed food, we made nutritional assessments of the following intakes: total energy (calories), carbohydrates, lipids, proteins, fiber, thiamine, riboflavin, niacin, pantothenic acid, vitamin B_6_, biotin, vitamin B_12_, C, D and E; and also minerals: calcium, phosphorus, magnesium, iron, zinc, iodine, selenium and fluoride. For nutrients presumed harmful (e.g., lipids, cholesterol), the opposite interpretation was applied; diet was considered inadequate if these limits were exceeded, and any intake below them was considered adequate.

### 2.6. Statistical Analysis

For the anthropometric measures, we compared the four categories (underweight, normoweight (healthy), overweight and obesity) between both genders (girls and boys) with frequency and percentage. We applied Bonferroni corrections to control for multiple comparisons. The EAR or AI cut-point method and the probabilistic approach were used to assess the risk of inadequate nutrient intakes. We ran a Student’s *t*-test to compare nutritional intakes in children with a different anthropometric status. For the dichotomous categorical variables, we compared the inadequacy of children’s diet to recommended intakes (recommendations met, not at risk *vs.* not met, at risk) by contingency tables, and using the χ^2^ test (or Fisher’s exact test, as and when appropriate) to assess statistical significance. The Shapiro-Wilk test was used to confirm assumptions of normality, linearity, homocedasticity and independence. All the *p* values were two-tailed and statistical significance was set at the conventional cut-off of *p* < 0.05. Data were entered into an Excel spreadsheet, using double-data entry to minimize the risk of errors, and then transferred to the IBM SPSS version 17.0 software (SPSS Inc., Chicago, IL, USA).

## 3. Results

### 3.1. Demographic Characteristics

#### 3.1.1. Children Aged 6–9 Years

The study sample included 710 schoolchildren made up of 372 girls (52.4%) and 338 boys (49.9%). Table 1 includes the characteristics of our sample group according to gender and the four anthropometric categories employed. The study sample’s mean age was 7.95 ± 1.12 years. Girls were heavier than boys, boys were taller, but no statistically significant gender differences were found.

The normoweight prevalence of the study sample was 53.1% (95% CI: 49.35–56.82), and it was higher for girls (53.49%, 95% CI: 48.28–58.65) than for boys (52.66%, 95% CI: 48.28–58.65), but no gender differences were found. Boys showed a higher underweight prevalence of 9.46% (95% CI: 6.56–13.10) *vs.* 7.25% (95% CI: 4.83–10.38) in girls, and also a higher obesity prevalence of 20.11% (95% CI: 15.97–24.79) *vs.* 18.01% (95% CI: 14.24–22.30), and a statistically significant difference between gender was observed (*p* = 0.045). When analyzing height compared to BMI, we observed that underweight boys (129.19 ± 8.41) and normoweight girls (129.39 ± 9.28) were shorter, with statistically significant differences for boys (*p* < 0.001) and girls (*p* = 0.004). A growing trend was also noted for height in both genders as the anthropometric category increased.

**Table 1:** Characteristics of the sample, according to BMI, in the 6–9 years old schoolchildren of both genders.

Variable	Total		Underweight		Normoweight		Overweight		Obesity		
	Mean	SD	Mean	SD	Mean	SD	Mean	SD	Mean	SD	*p* value
Both sexes	(n 710)	100%	(n 59)	8.3%	(n 377)	53.1%	(n 139)	19.6%	(n 135)	19.0%	
Age (years)	7.95	1.12	7.88	1.30	7.87	1.14	8.09	1.08	8.06	0.99	0.839
Weight (kg)	30.95	7.65	29.09	11.04	27.45	4.91	33.50	5.93	38.92	6.84	0.434
Height (cm)	130.95	8.94	129.76	8.68	129.34	8.68	132.58	9.29	134.29	8.24	0.684
Boys	(n 338)	100%	(n 32 )	9.46%	(n 178)	52.66%	(n 60 )	17.75%	(n 68 )	20.11%	
Age (years)	7.94	1.08	7.66	1.11	7.79	1.13	8.30	1.01	8.15	0.87	0.002
Weight (kg)	30.71	7.32	28.83	10.78	27.35	4.53	33.73	5.61	37.75	6.60	0.001
Height (cm)	131.28	8.47	129.19	8.41	129.28	7.98	133.48	8.96	134.63	7.81	0.001
Girls	(n 372)	100%	(n 27 )	7.25%	(n 199)	53.49%	(n 79)	21.23%	(n 67 )	18.01%	
Age (years)	7.96	1.16	8.14	1.48	7.94	1.14	7.94	1.11	7.97	1.11	0.830
Weight (kg)	31.16	7.95	29.40	11.53	27.54	5.23	33.33	6.19	40.10	6.91	0.001
Height (cm)	130.82	9.36	130.44	9.18	129.39	9.28	131.90	9.53	133.94	8.71	0.004
p (boys/girls) weight	0.434		0.845		0.708		0.695		0.045		
p (boys/girls) height	0.988		0.587		0.902		0.322		0.629		

Notes: SD: Standard Deviation; Means with different superscripts were statistically significant at *p* < 0.05 (Student’s *t*-test).

#### 3.1.2. Nutritional Characteristics

Table 2 presents nutritional characteristics of the sample according to BMI categories. When we analyzed nutrient intake in boys in accordance with the four anthropometric categories, we observed a statistically significant difference for the intakes of energy, niacin, vitamin D, phosphorus and selenium (*p* < 0.05). However for girls, no statistically significant differences were found for any type of nutrient intake. When we compared such intakes between boys and girls according to their anthropometric characteristics, we found statistically significant differences for the intakes of energy, carbohydrates, protein, lipids, riboflavin, niacin, vitamin B6, folic acid, calcium, phosphorus, magnesium, iron and selenium (*p* < 0.05).

### 3.2. Comparing Nutritional Intake According to DRIs

Table 3 summarizes the proportions of nutritional adequacy in all the children and in the four anthropometric categories. This study indicated intake which in all the children implied: (a) lower than recommended: biotin (inadequate deficit, 98.0%), fiber (inadequate deficit, 97.0%), flouride (inadequate deficit, 92.4%), vitamin D (inadequate deficit, 83.1%), zinc (inadequate deficit, 73.4%), iodine (inadequate deficit, 50.4%), vitamin E (inadequate deficit, 63.5%), folic acid (inadequate deficit, 37.1%), calcium (inadequate deficit, 30.2%), and iron (inadequate deficit, 14.8%); (b) all the children ate more than the recommended quantity of lipids (inadequate excess, 84.9%), proteins (inadequate excess, 66.6%) and cholesterol (inadequate excess, 17.2%).

**Table 2:** Nutritional characteristics of the sample according to BMI in children aged 6–9 years of both genders.

Nutrient	Boys (n 338)								*p* Value	Girls (n 372)								*p* Value	*p* Value Boy/Girl
	Underweight (n 32)		Normoweight (n 178)		Overweight (n 60)		Obesity (n 68)			Underweight (n 27)		Normoweight (n 199)		Overweight (n 79)		Obesity (n 67)			
	Mean	SD	Mean	SD	Mean	SD	Mean	SD		Mean	SD	Mean	SD	Mean	SD	Mean	SD		
Total energy (kcal/day)	1958.81	397.79	2176.76	403.81	2145.47	454.29	2204.24	498.64	0.049	1990.93	387.49	2072.61	396.74	2028.49	420.93	1971.45	329.22	0.268	0.001
Carbohydrates (g/day)	210.19	50.11	229.42	44.07	225.32	46.40	230.53	46.46	0.150	201.19	43.32	214.87	45.32	207.59	49.94	209.24	36.33	0.345	0.001
Protein (g/day)	83.14	23.25	88.95	20.91	84.98	19.49	92.72	24.78	0.106	84.08	27.20	83.24	19.09	82.23	22.50	80.46	19.01	0.697	0.003
Lipids (g/day)	84.57	20.80	96.20	22.56	96.62	28.22	97.12	28.84	0.057	90.84	23.48	94.16	23.12	92.68	22.84	87.28	21.71	0.198	0.045
Cholesterol (mg/day)	287.50	77.93	325.74	106.23	321.12	103.02	336.91	111.57	0.171	320.89	123.58	307.65	93.85	315.04	97.01	290.91	97.09	0.405	0.079
Fiber (g/day)	12.15	5.55	13.97	12.45	13.33	7.18	15.11	6.98	0.553	11.83	5.17	12.94	5.56	13.86	8.31	12.48	5.34	0.396	0.426
Thiamin (mg/day)	1.38	0.48	1.44	0.56	1.42	0.42	1.45	0.47	0.921	1.25	0.29	1.37	0.59	1.29	0.36	1.40	0.75	0.478	0.396
Riboflavin (mg/day)	1.71	0.44	1.98	0.68	1.98	0.57	1.83	0.48	0.053	1.70	0.48	1.77	0.54	1.71	0.52	1.71	0.54	0.740	0.001
Niacin (mg/day)	30.08	10.23	36.32	11.27	34.79	9.98	37.20	10.75	0,009	33.54	12.38	33.18	9.20	33.27	8.34	32.14	9.03	0.848	0.001
Pantothenic acid (mg/day)	5.17	1.53	5.60	1.49	5.33	1.53	5.60	1.61	0.351	5.41	1.43	5.39	1.33	5.19	1.28	5.34	1.35	0.715	0.549
Vit. B_6_ (mg/day)	1.81	0.69	2.16	0.82	2.08	0.68	2.08	0.60	0.108	1.91	0.70	1.94	0.66	1.92	0.52	1.84	0.74	0.758	0.006
Botin (µg/day)	27.27	9.13	28.52	8.27	26.57	7.22	27.27	8.45	0.374	26.69	8.25	26.75	8.51	26.79	8.69	26.53	10.11	0.998	0.563
Vit. B_12_ (µg/day)	5.29	2.67	5.97	3.53	6.56	4.71	6.63	3.27	0.248	5.17	3.22	6.03	3.89	5.94	3.35	5.70	2.84	0.656	0.461
Folic acid (µg/day)	226.28	58.49	250.67	104.38	251.86	60.03	244.77	81.85	0.530	219.70	73.79	230.49	76.00	223.77	69.47	218.16	87.28	0.652	0.001
Vit. C (mg/day)	96.45	42.77	105.89	54.96	113.82	52.94	108.04	55.51	0.513	101.21	40.52	97.11	44.86	95.21	43.98	90.242	47.78	0.659	0.074
Vit. A (µg/day)	343.03	154.78	546.45	623.14	587.78	852.04	472.17	295.09	0.223	420.15	299.21	480.90	630.37	399.81	233.06	413.10	297.87	0.568	0.189
Vit. D (µg/day)	2.38	1.45	3.50	3.14	2.76	1.99	3.68	2.74	0.046	2.57	2.40	3.13	2.40	2.87	2.06	2.96	3.04	0.656	0.080
Vit. E (µg/day)	6.66	1.98	7.72	3.18	7.58	2.65	8.43	3.66	0.062	7.59	3.00	7.53	2.68	7.97	2.82	7.04	2.43	0.229	0.072
Calcium (mg/day)	896.56	282.63	995.24	261.37	964.38	250.88	943.32	267.13	0.147	880.04	218.35	925.14	299.44	881.51	273.81	887.61	238.73	0.558	0.021
Phosphorus (mg/day)	1274.12	277.42	1457.02	366.31	1424.40	315.24	1468.22	380.60	0.048	1323.19	359.45	1363.77	340.98	1325.09	309.38	1306.04	315.93	0.585	0.001
Magnesium (mg/day)	258.58	68.82	281.53	87.99	279.98	72.73	288.13	84.85	0.421	256.85	67.29	270.57	69.32	262.57	68.34	248.15	58.87	0.117	0.019
Iron (mg/day)	11.66	4.08	13.84	6.16	13.40	4.64	13.41	4.02	0.178	12.12	4.28	12.36	4.04	12.21	3.83	11.94	4.46	0.905	0.011
Zinc (mg/day)	11.55	20.99	9.44	2.50	9.09	1.83	9.33	2.23	0.364	8.73	2.31	8.71	1.93	8.74	2.28	8.49	1.80	0.867	0.105
Iodine (mg/day)	84.39	21.30	104.36	61.42	94.82	25.40	120.70	157.29	0.166	96.45	28.89	95.88	35.57	90.35	24.48	88.56	26.11	0.283	0.032
Selenium (µg/day)	96.90	36.73	114.33	38.15	109.49	32.95	126.78	41.78	0.002	109.17	42.38	108.52	35.48	107.51	33.51	104.71	31.97	0.885	0.002
Fluoride (µg/day)	221.22	136.27	312.38	322.44	296.11	361.80	257.82	191.74	0.302	266.56	248.80	271.94	248.91	307.53	291.45	297.24	317.67	0.740	0.643

Notes: SD: Standard Deviation; Vit.: vitamin; *p* value < 0.5: was considered statistically significant (Student’s-test).

We identified statistically significant differences between the different anthropometric categories for BMI according to the DRIs in the intakes of carbohydrates (*p* < 0.049), riboflavin (*p* < 0.024), vitamin B_6_ (*p* < 0.001), vitamin D (*p* < 0.016) and iron (*p* < 0.017).

**Table 3:** Nutritional intake of the sample according to DRI and BMI in children aged ≥6 and <10 years.

Nutrient	DRIs	Total (n 710)		Underweight (n 59)		Normoweight (n 377)		Overweight (n 139)		Obesity (n 135)		*p* value
		n	%	n	%	n	%	n	%	n	%	
Carbohydrates (% TEV)	Under DRIs	673	94.8	56	94.9	357	94.7	133	95.7	127	94.1	0.049
	Met DRIs	37	5.2	3	5.1	20	5.3	6	4.3	8	5.9	
	Over DRIs	0	0	0	0	0	0	0	0	0	0	
Protein(% TEV)	Under DRIs	0	0	0	0	0	0	0	0	0	0	0.157
	Met DRIs	237	33.4	19	32.2	131	34.7	50	36.0	37	27.4	
	Over DRIs	473	66.6	40	67.8	246	65.3	89	64.0	98	72.6	
Lipids ^a^ (% TEV)	Under DRIs	15	2.1	3	5.1	6	1.6	1	0.7	5	3.7	0.174
	Met DRIs	92	13.0	4	6.8	47	12.5	22	15.8	19	14.1	
	Over DRIs	603	84.9	52	88.1	324	85.9	115	82.7	111	82.2	
Cholesterol ^a^ (mg/1000 kcal) ^b^	Under DRIs	200	28.2	13	22.0	115	30.5	33	23.7	39	28.9	0.691
	Met DRIs	388	54.6	36	61.0	201	53.3	79	56.8	72	53.3	
	Over DRIs	122	17.2	10	16.9	61	16.2	27	19.4	24	17.8	
Fiber (g/day)	Under DRIs	689	97.0	57	96.6	368	97.6	135	97.1	129	95.6	0.680
	Met DRIs	21	3.0	2	3.4	9	2.4	4	2.9	6	4.4	
Thiamin (mg/day)	Under DRIs	42	5.9	7	11.9	22	5.8	6	4.3	7	5.2	0.210
	Met DRIs	668	94.1	52	88.1	355	94.2	133	95.7	128	94.8	
Riboflavin (mg/day)	Under DRIs	55	7.7	10	16.9	22	5.8	13	9.35	10	7.4	0.024
	Met DRIs	655	92.3	49	83.1	355	94.2	126	90.65	125	92.6	
Niacin (mg/day)	Under DRIs	1	0.1	1	1.7	0	0	0	0	0	0	-
	Met DRIs	709	99.9	58	98.3	377	100	139	100	135	100	
Pantothenic acid (mg/day)	Under DRIs	7	1.0	2	0.5	1	1.7	2	1.4	2	1.5	-
	Met DRIs	703	99.0	375	99.5	58	98.3	137	98.6	133	98.5	
Vit. B_6_ (mg/day)	Under DRIs	100	14.1	13	22.0	53	14.1	15	10.8	1	14.1	0.001
	Met DRIs	610	85.9	46	78.0	324	85.9	124	89.2	116	85.9	
Botin (µg/day)	Under DRIs	14	2.0	58	98.3	371	98.4	136	97.8	130	96.3	0.532
	Met DRIs	696	98.0	1	1.7	6	1.6	3	2.2	5	3.7	
Vit. B_12_ (µg/day)	Under DRIs	2	0.3	0	0	0	0	1	0.7	1	0.7	-
	Met DRIs	708	99.7	59	100	377	100	138	99.3	134	99.3	
Folic acid (µg/day)	Under DRIs	263	37.1	25	42.4	137	36.3	45	32.4	56	41.5	0.356
	Met DRIs	447	62.9	34	57.6	240	63.7	94	67.6	79	58.5	
Vit. C (mg/day)	Under DRIs	115	16.2	9	15.3	63	16.7	18	12.9	25	18.5	0.630
	Met DRIs	595	83.8	50	84.7	314	83.3	121	87.1	110	81.5	
Vit. A (µg/day)	Under DRIs	68	9.6	6	10.2	31	8.2	17	12.2	14	10.4	0.562
	Met DRIs	642	90.4	53	89.6	346	91.8	122	87.8	121	89.6	
Vit. D (µg/day)	Under DRIs	591	83.1	54	91.5	302	80.1	125	89.9	110	81.51	0.016
	Met DRIs	119	16.9	5	8.5	75	19.9	14	10.1	25	18.5	
Vit. E (mg/day)	Under DRIs	451	63.5	45	76.3	237	62.9	81	58.3	92	68.1	0.098
	Met DRIs	259	36.5	14	23.7	140	37.1	58	41.7	47	31.9	
Calcium (mg/day)	Under DRIs	214	30.2	19	32.8	107	28.4	46	33.1	42	31.1	0.710
	Met DRIs	495	69.8	39	67.2	270	71.6	93	66.9	93	68.9	
Phosphorus (mg/day)	Under DRIs	5	0.7	1	1.7	3	0.8	0	0.0	1	0.7	-
	Met DRIs	705	99.3	58	98.3	374	99.2	139	100	134	99.3	
Magnesium (mg/day)	Under DRIs	43	6.1	3	5.1	19	5.1	10	7.2	11	8.3	0.529
	Met DRIs	664	93.9	56	94.9	357	94.9	129	92.8	122	91.7	
Iron (mg/day)	Under DRIs	105	14.8	17	28.8	52	13.8	19	13.7	17	12.2	0.017
	Met DRIs	605	85.2	42	71.2	325	86.2	120	86.3	118	87.8	
Zinc (mg/day)	Under DRIs	521	73.4	48	81.4	275	72.9	100	71.9	98	72.6	0.542
	Met DRIs	189	26.6	11	18.6	102	27.1	39	28.1	37	27.4	
Iodine (µg/day)	Under DRIs	358	50.4	34	57.6	175	46.4	75	53.9	73	51.1	0.168
	Met DRIs	352	49.6	25	42.4	202	53.6	64	46.1	62	45.9	
Selenium (µg/day)	Under DRIs	0	0	0	0	0	0	0	0	0	0	-
	Met DRIs	710	100	59	100	377	100	139	100	135	100	
Fluoride (µg/day)	Under DRIs	669	94.2	58	98.3	352	93.4	131	94.2	128	94.8	0.493
	Met DRIs	41	5.8	1	1.7	25	6.6	8	5.8	7	5.2	

Notes: Vit.: vitamin, TEV: total energy value, DRIs: EAR: carbohydrates (50%–60% TEV), proteins (10%–15% TEV), lipids (30%–35% TEV), thiamin (0.8 mg/day), riboflavin (1.2 mg/day), niacin (12 mg/day), vit B_6_ (1.4 mg/day), biotin (12 µg/day), vit B_12_ (1.5 µg/day), folic acid (200 µg/day), vit. C (55 mg/day), vit. A (400 µg/day), vit D (5 µg/day), vit E (8 mg/day), calcium (800 mg/day), phosphorus (700 mg/day), iron (9 mg/day), zinc (10 mg/day), iodine (90 µg/day), selenium (30 µg/day) and AI: fiber (25 mg/day), panthothenic acid (3 mg/day), magnesium (180 mg/day), fluoride (1000 µg/day). *p* value < 0.05: was considered statistically significant (Student’s *t*-test). ^a^ Intakes failed to meet recommendations if they under DRIs, except for total fats and cholesterol, for which inadequate intakes were those over DRIs or nutritional target for Spanish people, respectively. ^b^ The DRI for cholesterol was not determinable. Instead the target for the Spanish population of 100 mg/1000 kcal was considered.

## 4. Discussion

Anthropometry remains one of the most widely used methods for assessing and monitoring health status, nutritional status, as well as child growth in individuals and communities [41]. The present study identified that 53.1% of the population of Valencian schoolchildren was normoweight and 38.6% had excess body weight (19.6% overweight and 19.0% obesity). Being overweight is a worldwide problem that affects developed and developing countries alike. When we compared different Spanish studies conducted in recent decades, we found an alarming growing trend. According to data published in 2006 by the ENSE [42], the prevalence of overweight in children was 21.43% and 15.38% for obesity, as opposed to ENSE published in 2013 [43], with 23.9% overweight and 16.0% obesity. Locally conducted health surveys (the Valencian Community) [44] showed that overweight prevalence was 18.0% overweight and 22.3% for obesity, which means that 4 children in every 10 were overweight or obese, and clearly indicates the progressive increase in the childhood overweight status in our country. This overweight-obesity pattern is similar to that described in other areas in Spain [45,46,47]. European studies have shown a similar percentage of overweight and obesity [48,49,50]. Excess body weight (overweight and obesity) is a multifactorial disorder whose ethiopathogeny implies genetic, metabolic, psychosocial and environmental factors. However, the speed with which their prevalences have increased apparently relates more with environmental factors; e.g., healthy eating habits [51].

Our findings suggest that children aged 6–9 years show scant compliance with the nutritional goals set by the DRIs of the Spanish population [38,39] for biotin, fiber, flouride, vitamin D, zinc, iodine, vitamin E, folic acid, calcium and iron. Excessive lipids, proteins and cholesterol intakes were observed in both sexes. Unexpectedly there were normoweight children who represented the “healthy” category, but did not present proper intakes for their age and gender as they were lower than those recommended. For instance for the normoweight group, a higher micronutrients intake was obtained than in the overweight and obesity categories. It is worth stressing, however, that intake estimates below recommendations did not indicate nutrient deficiencies as recommended intakes far exceeded the mean requirement. However, they are useful for indicating potential deficiencies, which will become greater the larger the differences between those calculated based on real and recommended intakes (DRI). True deficiency statuses should be diagnosed by other means, especially through biochemical analyses [52,53,54].

This study used the nutritional adequacy values from the latest SENC [38] and FESNAD [39] reviews; both institutions assess nutrition in Spain. The values we employed were taken in accordance with age, which we did not separate per gender because the same levels were presented for our study sample’s age group. We found that the DRIs values that covered the nutritional requirements of 50% of the healthy population were the so-called EAR, while the IA are based on the requirements identified experimentally in a healthy population. We used the EAR for carbohydrates, protein, lipids, thiamin, riboflavin, niacin, vitamin B_6,_ biotin, vitamin B_12_, folic acid, vitamin C, vitamin A, vitamin D, vitamin E, calcium, phosphorus, iron, zinc, iodine and selenium, and the AI parameter for fiber, panthothenic acid, magnesium and fluoride because the EAR for these nutrients have not been determined. We considered this difference in the known reference value when we interpreted the results of nutritional deficiencies.

A diet adequate from a nutritional viewpoint implies a balanced nutritional diet, and that the undesirable quantities of saturated fats, trans-fatty acids or sugars, among others, are minimum, which are related to highly prevalent childhood diseases, such as obesity, hypercholesterolemia or dental caries [52]. Therefore, if child feeding is correct, and if diet nutritional quality is adequate and varied, it will have a direct influence on growth and development.

Energy intake was adequate in most children, and all the anthropometric categories increased, except the obese girls, whose mean intake was similar to that of the underweight category. This might be due to families controlling their eating habits as they are aware that overweight children are at higher risk of becoming obese in adulthood [55]. The observed higher energy intake in boys is consistent with the results of previous studies, which have reported significantly higher energy intake in boys compared to girls. This result was similar to those reported in several Spanish studies [46,47,56]. The intake of carbohydrates and fiber was below that recommended, and this finding coincides with other authors [57,58,59,60,61]. These macronutrients are key nutrients for various body functions, and their low intake may be due children’s general rejection of vegetable and cereals. Of all the schoolchildren we studied, 84.5% presented a lipids intake over the DRIs. The importance of acting with these children stems from the high risk they have of developing degenerative diseases (cardiovascular and obesity) from eating too many lipids, and not just in the short term, but also in adulthood [2,18]. In turn, 17.2% of the schoolchildren presented a value over the DRIs for cholesterol as their intake of monosaccharides and disaccaharides was high [18,62]. Thus reducing their sucrose intake is recommended [1]. Compared with other studies, the total protein intake we observed herein (regardless of it being of animal or plant origin) was lower than in studies done in Poland [2] and Portugal [56]. The high protein intake of our study may be due to most proteins being of animal origin (including proteins from meat, fish, eggs and milk), which can imply early puberty onset in the short term [63] and a long-term risk of diabetes [64].

The mean intakes of all the studied vitamins, except biotin, folic acid, vitamin D and vitamin E, were adequate. The action of biotin and folic acid is relevant because both these micronutrients are inversely related with the plasma homocysteine concentration, which is linked to a higher risk of developing cardiovascular diseases [40]. This is mainly due to children’s general rejection of fish, which could also justify the low vitamin D intake observed herein, with similar values to those found in other studies [52,65]. It should be emphasized that the normoweight category was that with the best mean intake. Low vitamin D intake is clinically associated with adverse health outcomes, including growth retardation, increased risk of autoimmune disease, and delayed dentition or bone deformities through inadequate calcification [66]. Vitamin D is synthesized as a result of exposure to solar ultraviolet-B irradiation [67,68,69], and the climate conditions of the Valencian Community could compensate this shortage. In the same way, vitamin E, n antioxidant present in the basic food items of the Mediterranean diet such as vegetable oils, nuts and green leafy vegetables [70], showed deficient intakes for half the study groups, but higher intakes in the overweight category.

Regarding minerals, even though the majority of the sample reached the DRI for calcium, a small part of the study sample did not. This mineral, which is present in milk, is essential in childhood, and its deficit dietary intake is involved in bone resorption mediated by the parathyroid hormone (PTH), which causes reduced bone mass and osteoporosis in adulthood [58]. Similarly zinc, present in meat, fish, poultry, milk and its by-products, provides 80% of total diet zinc [71]. It was deficient in all the anthropometric categories. This low intake is likely not that important since the whole sample were found to eat protein-rich diets. Finally, iodine values were similar for all the categories, which were all below the DRI, and did not meet 75% of the recommendation. Low iodine intake in children is clinically associated with higher prevalence of thyroid nodular diseases in adulthood [72]. A low iodine intake can be justified by children eating very few marine food items, whose rejection is widespread [73,74].

Suitable anthropometry in a child population is not necessarily a synonym of suitable food intake. Special attention should be paid to the observed nutrient deficiencies, and their intake should be actively reinforced from primary care and schools since such nutrients act directly on child growth and development. Intake of certain foods can be improved by setting up Food Education Programs to encourage healthy eating habits, to promote higher daily intakes of fish, fruit and vegetables, and to eat a decent breakfast. Parents’ education and socio-economic position probably influence children’s eating habits as they either facilitate or restrict understanding nutritional information and fulfilling nutritional recommendations. The school environment, along with family and community environments, are also the most influential educational areas where healthy eating and life style habits are acquired. Attitudes to be taken by schools in terms of nutritional aspects should be intrinsically exemplified to satisfy their educational purpose, and to consequently help avoid excessive body weight (overweight and obesity) in children [51].

### Study Limitations

In our child study, the data we had available were insufficient to establish a clear relationship between eating patterns and anthropometric status. Future studies should attempt to clarify this possible relationship. Although the Spanish dietary recommendations for the studied age group (6–9 years) are the same for boys and girls, this could be a weakness. Nor did we have information about arm circumference or triceps skinfold to enable us to know if fat was peripheral or central.

We believe that our study offers strong internal validity given the low attrition rate obtained. We are confident that the self-reported information employed for the nutrition assessment is good quality. Parents and schools were very interested in the study, and were extensively trained and supported to complete food records. They were also followed up over the same period. We understand that these factors compensate for limitations in our generalizability and external validity.

## 5. Conclusions

Our finding suggests that nutritional intervention and educational strategies are needed in this population of Spanish children to promote healthy eating and to correct inadequacies. Updating Spanish food composition is necessary to ensure reliable precise estimates of nutrient intake. The present study provides a baseline for future intervention programs to prevent children from suffering overweight and obesity problems based on the relevance of acquiring suitable eating habits.

## References

[1] FAO/WHO (2003). Diet, Nutrition and the Prevention of Chronic Diseases. http://www.who.int/dietphysicalactivity/publications/trs916/en/gsfao_introduction.pdf.

[2] Merkiel S. (2014). Dietary intake in 6-year-old children from southern Poland: Part I—Energy and macronutrient intakes. BMC Pediatr..

[3] The United Nations Children’s Fund La Desnutricion Infantil. Causas, Consecuencias y Estrategias Para su Prevencion y Tratamiento. https://www.unicef.es/sites/www.unicef.es/files/Dossierdesnutricion.pdf.

[4] Gibson E.L., Kreichauf S., Wildgruber A., Vögele C., Summerbell C.D., Nixon C., Moore H., Douthwaite W., Manios Y., ToyBox-Study Group (2012). A narrative review of psychological and educational strategies applied to young children’s eating behaviours aimed at reducing obesity risk. Obes. Rev..

[5] Lobstein T., Baur L., Uauy R. (2004). Obesity in children and young people: A crisis in public health. Obes. Rev..

[6] Must A., Strauss R.S. (1999). Risks and consequences of childhood and adolescent obesity. Int. J. Obes. Relat. Metab. Disord..

[7] McCrindle B.W. (2015). Cardiovascular consequences of childhood obesity. Can. J. Cardiol..

[8] Maunder E.M., Nel J.H., Steyn N.P., Kruger H.S., Labadarios D. (2015). Added sugar, macro- and micronutrient intakes and anthropometry of children in a developing world context. PLoS ONE.

[9] Mamun A.A., Finlay J.E. (2014). Shifting of undernutrition to overnutrition and its determinants among women of reproductive ages in the 36 low to medium income countries. Obes. Res. Clin. Pract..

[10] Mendez M.A., Monteiro C.A., Popkin B.M. (2005). Overweight exceeds underweight among women in most developing countries. Am. J. Clin. Nutr..

[11] Prentice A.M. (2006). The emerging epidemic of obesity in developing countries. Int. J. Epidemiol..

[12] Ziraba A.K., Fotso J.C., Ochako R. (2009). Overweight and obesity in urban Africa: A problem of the rich or the poor?. BMC Public Health.

[13] Kelishadi R., Azizi-Soleiman F. (2014). Controlling childhood obesity: A systematic review on strategies and challenges. J. Res. Med. Sci..

[14] St-Onge M.P., Keller K.L., Heymsfield S.B. (2003). Changes in childhood food consumption patterns: A cause for concern in light of increasing body weights. Am. J. Clin. Nutr..

[15] Reilly J.J., Methven E., McDowell Z.C., Hacking B., Alexander D., Stewart L., Kelnar C.J. (2003). Health consequences of obesity. Arch. Dis Child..

[16] Barlow S.E. (2007). Expert committee recommendations regarding the prevention, assessment, and treatment of child and adolescent overweight and obesity: Summary report. Pediatrics.

[17] FAO/WHO Salud de la Madre, el Recién Nacido, del Niño y del Adolescente. http://www.who.int/maternal_child_adolescent/topics/child/malnutrition/es/.

[18] Bogin B., Azcorra H., Wilson H.J., Vázquez-Vázquez A., Avila-Escalante M.L., Castillo-Burguete M.T., Varela-Silva I., Dickinson F. (2014). Globalization and children’s diets: The case of Maya of Mexico and Central America. Anthropol. Rev..

[19] Sawaya A.L., Martins P.A., Baccin-Martins V.J., Florêncio T.T., Hoffman D., do-Carmo P., Franco M., das-Neves J. (2009). Malnutrition, long-term health and the effect of nutritional recovery. Nestle Nutr. Workshop Ser. Pediatr. Program..

[20] Ogden C.L., Flegal K.M., Carroll M.D., Johnson C.L. (2002). Prevalence and trends in overweight among US children and adolescents, 1999–2000. JAMA.

[21] Instituto Nacional de Salud Pública Encuesta Nacional de Salud y Nutrición 2012. http://ensanut.insp.mx/informes/Yucatan-OCT.pdf.

[22] Serra L., Ribas L., Aranceta J., Pérez C., Saavedra P., Peña L. (2003). Obesidad infantil y juvenil en Espana. Resultados del Estudio enKid (1998–2000). Med. Clin..

[23] Ortega R.M. Estudio ALADINO. Estudio de Vigilancia del Crecimiento, Alimentación, Actividad Física, Desarrollo Infantil y Obesidad en España, 2011. http://www.observatorio.naos.aesan.msssi.gob.es/docs/docs/documentos/estudio_ALADINO.pdf.

[24] Miqueleiz E., Lostao L., Ortega P., Santos J.M., Astasio P., Regidor E. (2014). Socioeconomic pattern in unhealthy diet in children and adolescents in Spain. Aten. Primaria.

[25] Ministerio de Sanidad, Servicios Sociales e Igualdad (MSSSI) (2005). Estrategia NAOS. http://www.naos.aesan.msssi.gob.es/naos/ficheros/estrategia/estrategianaos.pdf.

[26] Majem S.L., Bartrina A.J., Barba R.L., Monroy S.M., Rodrigo P.C. (2003). Crecimiento y Desarrollo: Dimensión Alimentaria y Nutricional.

[27] Hernández-Rodríguez M. (2000). El patrón de crecimiento. Tratado de Endocrinología Pediátrica y de la Adolescencia.

[28] World Health Organization (2006). Child Growth Standards: Length/Height-for-Age, Weight-for-Age, Weight-for-Length, Weight-for-Height and Body Mass Index for Age. http://www.who.int/childgrowth/standards/Technical_report.pdf.

[29] Onis M., Onyango A.W., Borghi E., Siyam A., Nishida C., Siekmann J. (2007). Development of a WHO growth reference for school-aged children and adolescents. Bull. World Health Organ..

[30] World Health Organization OMS Anthro, a Software for Assessing Growth and Development of the World’s Children (Version 3.2.2). http://www.who.int/childgrowth/software/es/.

[31] Centers for Disease Control and Prevention Defining Childhood Overweight and Obesity. http://www.cdc.gov/obesity/childhood/defining.html.

[32] Barrett-Connor E. (1991). Nutrition epidemiology: How do we know what they ate?. Am. J. Clin. Nutr..

[33] Institute of Medicine (IOM) (2001). Dietary Reference Intakes: Applications in Dietary Assessment.

[34] Ortega R.M., Lopez A.M., Andrés P., Requejo A.M., Aparicio A., Molinero L.M. (2012). DIAL Programa Para la Evaluación de Dietas y Gestión de Datos de Alimentación.

[35] Institute of Medicine (IOM) (2000). Dietary Reference Intakes: Applications in Dietary Assessment.

[36] Institute of Medicine (IOM) (2003). Dietary Reference Intakes: Applications in Dietary Planning.

[37] Murphy S.P., Barr S.I. (2011). Practice paper of the American Dietetic Association: Using the dietary reference intakes. J. Am. Diet. Assoc..

[38] Sociedad Española de Nutrición Comunitaria (SENC) (2001). Objetivos nutricionales para la poblacion española. Span. J. Community Nutr..

[39] Federación Española de Sociedades de Nutrición; Alimentación y Dietética (FESNAD) (2010). Ingestas dietéticas de referencia (IDR) para la población española. Act. Diet..

[40] Carriquiry A.L. (1999). Assessing the prevalence of nutrient inadequacy. Public Health Nutr..

[41] Arija V., Pérez-Rodrigo C., Martínez-de-Vitoria E., Ortega R.M., Serra-Majem L., Ribas L., Aranceta J. (2015). Dietary intake and anthropometric reference values in population studies. Nutr. Hosp..

[42] Ministerio de Sanidad Servicios Sociales e Igualdad, Instituto Nacional de Estadística (2006). Encuesta Nacional de Salud de España. http://www.msssi.gob.es/estadEstudios/estadisticas/encuestaNacional/encuestaIndice2006.htm.

[43] Ministerio de Sanidad Servicios Sociales e Igualdad, Instituto Nacional de Estadística Encuesta Nacional de Salud de España 2011–2012. http://www.msssi.gob.es/estadEstudios/estadisticas/encuestaNacional/encuestaNac2011/encuestaResDetall2011.htm.

[44] Generalitat Valenciana (2010). Encuesta de la Salud de la Comunidad Valenciana. http://www.san.gva.es/web/sdg-i-d-i/documento-fraccionado-encuesta-salud.

[45] Gulías-González R., Martínez-Vizcaíno V., García-Prieto J.C., Díez-Fernández A., Olivas-Bravo A., Sánchez-López M. (2014). Excess of weight, but not underweight, is associated with poor physical fitness in children and adolescents from Castilla-La Mancha, Spain. Eur. J. Pediatr..

[46] Rodriguez-Artalejo F., Rodrıguez-Artalejo F., Garces C., Gorgojo L., Lopez E., Martın-Moreno J.M., Benavente M., del Barrio J.L., Rubio R., Ortega H. (2002). Dietary patterns among children aged 6–7 years in four Spanish cities with widely differing cardiovascular mortality. Eur. J. Clin. Nutr..

[47] Pérez-Farinós N., López-Sobaler A.M., Dal-Re M.Á., Villar C., Labrado E., Robledo T., Ortega R.M. (2013). The ALADINO study: A national study of prevalence of overweight and obesity in Spanish children in 2011. Biomed. Res. Int..

[48] Cadenas-Sanchez C., Nyström C.D., Sanchez-Delgado G., Martinez-Tellez B., Mora-Gonzalez J., Risinger A.S., Ruiz J.R., Ortega F.B., Löf M. (2015). Prevalence of overweight/obesity and fitness level in preschool children from the north compared with the south of Europe: An exploration with two countries. Pediatr. Obes..

[49] Maffeis C., Tommasi M., Tomasselli F., Spinelli J., Fornari E., Scattolo N., Marigliano M., Morandi A. (2015). Fluid intake and hydration status in obese *vs.* normal weight children. Eur. J. Clin. Nutr..

[50] Smetanina N., Albaviciute E., Babinska V., Karinauskiene L., Albertsson-Wikland K., Petrauskiene A., Verkauskiene R. (2015). Prevalence of overweight/obesity in relation to dietary habits and lifestyle among 7–17 years old children and adolescents in Lithuania. BMC Public Health.

[51] Travé D.T., Victoriano G.F., Grupo Colaborador de Navarra (2013). Natural evolution of excess body weight (overweight and obesity) in children. An. Pediatr..

[52] Serra-Majem L., Ribas-Barba L., Pérez-Rodrigo C., Bartrina J.A. (2006). Nutrient adequacy in Spanish children and adolescents. Br. J. Nutr..

[53] GIBSON (1990). Evaluation of Nutrient Intake Data Principles of Nutritional Assessment.

[54] Henríquez-Sánchez P., Díaz-Romero C., Rodríguez-Rodríguez E., López-Blanco F., Alvarez-Leon E., Díaz-Cremades J., Pastor-Ferrer M.C., Serra-Majem L. (2000). Evaluación bioquímica del estado nutricional de la población canaria (1997–1998). Arch. Latinoam. Nutr..

[55] Singh A.S., Mulder C., Twisk J.W.R., van-Mechelen W., Chinapaw M.J.M. (2008). Tracking of childhood overweight into adulthood: A systematic review of the literature. Obes. Rev..

[56] Moreira P., Padez C., Mourão I., Rosado V. (2005). Dietary calcium and body mass index in Portuguese children. Eur. J. Clin. Nutr..

[57] Ruiz-Roso B., Pérez-Olleros L. (2010). Avance de resultados sobre el consumo de fibra en España y beneficios asociados a la ingesta de fibra insoluble. Rev. Esp. Nutr. Comunitaria.

[58] Huang J.Y., Qi S.J. (2015). Childhood obesity and food intake. World J. Pediatr..

[59] Khan N.A., Raine L.B., Drollette E.S., Scudder M.R., Kramer A.F., Hillman C.H. (2015). Dietary fiber is positively associated with cognitive control among prepubertal children. J. Nutr..

[60] Kolahdooz F., Butler J.L., Christiansen K., Diette G.B., Breysse P.N., Hansel N.N., McCormack M.C., Sheehy T., Gittelsohn J., Sharma S. (2015). Food and nutrient intake in African American children and adolescents aged 5 to 16 years in Baltimore City. J. Am. Coll. Nutr..

[61] Storey M., Anderson P. (2014). Income and race/ethnicity influence dietary fiber intake and vegetable consumption. Nutr. Res..

[62] Niinikoski H., Ruottinen S. (2012). Is carbohydrate intake in the first years of life related to future risk of NCDs?. Nutr. Metab. Cardiovasc. Dis..

[63] Cheng G., Buyken A.E., Shi L., Karaolis-Danckert N., Kroke A., Wudy S.A., Degen G.H., Remer T. (2012). Beyond overweight: Nutrition as an important lifestyle factor influencing timing of puberty. Nutr. Rev..

[64] Sluijs I., Beulens J.W., van der A D.L., Spijkerman A.M.W., Grobbee D.E., van der Schouw Y.T. (2010). Dietary intake of total, animal, and vegetable protein and risk of type 2 diabetes in the European prospective investigation into cancer and nutrition (EPIC)-NL study. Diabetes Care.

[65] Osowski C.P., Lindroos A.K., Barbieri H.E., Becker W. (2015). The contribution of school meals to energy and nutrient intake of Swedish children in relation to dietary guidelines. Food Nutr. Res..

[66] Zerofsky M., Ryder M., Bhatia S., Stephensen C.B., King J., Fung E.B. (2015). Effects of early vitamin D deficiency rickets on bone and dental health, growth and immunity. Matern. Child. Nutr..

[67] Cutillas-Marco E., Fuertes-Prosper A., Grant W.B., Morales-Suárez-Varela M.M. (2012). Vitamin D deficiency in South Europe: Effect of smoking and aging. Photodermatol. Photoimmunol. Photomed..

[68] Llopis-González A., Rubio-López N., Pineda-Alonso M., Martín-Escudero J.C., Chaves F.J., Redondo M., Morales-Suarez-Varela M. (2015). Hypertension and the fat-soluble vitamins A, D and E. Int. J. Environ. Res. Public Health.

[69] Grant W.B., Holick M.F. (2005). Benefits and requirements of vitamin D for optimal health: A review. Altern. Med. Rev..

[70] Kim Y.N., Cho Y.O. (2015). Vitamin E status of 20- to 59-year-old adults living in the Seoul metropolitan area of South Korea. Nutr. Res. Pract..

[71] Sandstead H.H., Freeland-Graves J.H. (2014). Dietary phytate, zinc and hidden zinc deficiency. J. Trace Elem. Med. Biol..

[72] Vandevijvere S., Dramaix M., Moreno-Reyes R. (2012). Does a small difference in iodine status among children in two regions of Belgium translate into a different prevalence of thyroid nodular diseases in adults?. Eur. J. Nutr..

[73] Campos R.D., Barreto I.D., Maia L.R., Rebouças S.C., Cerqueira T.L., Oliveira C.A., Santos C.A., Mendes C.M., Teixeira L.S., Ramos H.E. (2015). Iodine nutritional status in Brazil: A meta-analysis of all studies performed in the country pinpoints to an insufficient evaluation and heterogeneity. Arch. Endocrinol. MeTable.

[74] Katzen-Luchenta J. (2007). The declaration of nutrition, health, and intelligence for the child-to-be. Nutr. Health.

